# Understanding South Korean women workers’ career transition experiences: using the career decision tree model

**DOI:** 10.3389/fpsyg.2024.1273241

**Published:** 2024-04-02

**Authors:** Namhee Kim, Kyung Nam Kim, Pyounggu Baek

**Affiliations:** ^1^Department of Education, Ewha Womans University, Seoul, Republic of Korea; ^2^College of General Education, Kookmin University, Seoul, Republic of Korea

**Keywords:** South Korean women, career transitions, career history, out-out, women’s career

## Abstract

**Introduction:**

Relatively little research has explored non-Western women workers and their career transitions within their unique cultural contexts. Thus, more context-sensitive approaches to women’s career trajectories are needed.

**Methods:**

Based on Bian and Wang’s Career Decision Tree Model (2019) as a conceptual framework, the reasons for South Korean women workers’ career transitions and influencing factors were explored using a qualitative approach with in-depth interviews with 35 South Korean women workers at various career stages.

**Results and Discussion:**

Their main motive of career transitions was difficulty maintaining their physical and mental health, which stemmed from their demanding work life. A typical issue, the burden of child rearing and family responsibilities, was also reported, but it was not the primary reason for their career transitions. Instead, the women workers often mentioned these responsibilities along with other reasons. Other reasons were unresolved career interests and expectations associated with their lack of career goals and preparation prior to joining the labor market. These factors led to significant changes in women’s values and priorities along their career path, which finally triggered a decision to make a career transition. South Korean socio-cultural characteristics embedded in the South Korean women’s personal and organizational lives provide insights on how to interpret the findings. Although on the surface some of our findings appeared to confirm previous studies on women’s career transitions in Western-based literature, noteworthy differences were discovered when delving deeper into women’s career transitions in the South Korean context.

## 1 Introduction

As women’s participation in economic activity has increased, their careers have gained considerable attention from researchers over the past few decades (e.g., Powell and Mainiero, 1992; Mainiero and Sullivan, 2006; Cabrera, 2007; Kossek et al., 2017; Elley-Brown et al., 2018; Afiouni and Karam, 2019; Lafkas et al., 2023). Women’s careers have been described as non-linear patterns reflecting demands that are not work-related but rather socially and culturally prescribed (Fapohunda, 2014; Gardner et al., 2021). In contrast, the traditional career progression of males has shown upward and linear patterns (Evers and Sieverding, 2014; Lyons et al., 2015). Compared to men’s patterns, women often have less freedom to pursue the career development they desire (Powell and Mainiero, 1992; Bayeh, 2016; Bao and Tian, 2022).

Career transition is defined as an extrinsic process of changing jobs or professions, which often includes an intrinsic aspect (Louis, 1980). Understanding the extrinsic aspects of careers requires objective measurements including workers’ salary, benefits, and position. In contrast, intrinsic aspects reflect workers’ subjective perceptions of the invisible rewards of a career such as inherent interest in the work, purposeful work, and the opportunity for growth. A career transition can include two aspects: a change in one’s objective role or behavior and one’s personal orientation toward the role or relationships within their job (Hirschi and Koen, 2021). Therefore, it is necessary to understand how both extrinsic and intrinsic career aspects interplay when researching career transitions.

Many scholars in mainstream research focusing on women’s career transitions have emphasized the prevalence and surface reasons for their career transitions (Maxwell and Broadbridge, 2014; Lerchenmueller and Sorenson, 2018). Personal reasons for a career transition include family situations and responsibilities (e.g., child-rearing, elder care, and relocation following their spouse), and women’s pursuit of work-life balance (Hill et al., 2014; Sari et al., 2021). These situations act as pulling-in factors that can lead women to make a career transition. Zimmerman and Clark (2016) identified several “family factors that pull women away from their work to stay at home” (p. 620). Stone and Lovejoy (2004) also stated that women’s decision to opt-out of working is influenced by the pull of their husbands, which includes lack of support with parenting from their husband, and a preference for the wife to stay home. Patrick et al. (2016) also reported that the burden of family responsibilities such as taking care of young children made some married women withdraw from organizations and choose to be self-employed.

Beyond personal reasons, the literature has also discussed a wider scope of factors that push women out of the workplace (Hewlett, 2008). For example, Kossek et al. (2017) investigated whether women opt out or are pushed out of the workplace by analyzing individual and organizational factors related to equality in women’s careers. Hewlett (2008) characterized these push factors as the main features of the work environment. These factors are especially evident in organizations with a gendered organizational culture (Stone and Lovejoy, 2004; Cahusac and Kanji, 2014; Xie and Zheng, 2022), and work-related pushed out factors such as workplace inflexibility, long working hours, and high volume of work (Lim and Mohd Rasdi, 2019). According to Shropshire and Kadlec (2012) and Carless and Arnup (2011), work stress is a clear push factor leading to turnover, and women workers may be “squeezed out” (Metz, 2011, p. 287) of their organizations due to unwelcoming organizational factors.

However, women’s career transitions are not fully explained by the simple dichotomy of push vs. pull factors but include the dynamic interplay among personal, family, organizational, cultural, and other factors (Uludag et al., 2022). Thus, a career decision model for women needs to include complex issues that affect women’s career decisions such as the societal culture and norms, industrial culture and norms, organizational culture and strategies, national family policies, as well as the outcomes of women’s life events and individual career decisions (Bian and Wang, 2019). However, to date, relatively little research has explored non-Western women workers and their career transitions within their unique cultural contexts (Behery et al., 2017). Thus, more context-sensitive approaches to women’s career trajectories are needed. This study responds to this need by applying Bian and Wang’s (2019) career decision tree model (CDTM), grand framework to understand women’s career decisions in South Korea (hereafter, Korea).

Based on the CDTM as a conceptual framework, the purpose of this paper is to explore the career transition experiences of a group of Korean women workers to address the following research questions:

RQ1: What causes Korean women workers’ career transitions?

RQ2: How do various contextual factors affect their career transitions?

This study intends to fill the research gap in the mainstream research on women’s careers in terms of research focus, context, and methods. First, this study focuses on women’s career transitions at various career stages including both extrinsic and intrinsic aspects. By collecting circumstantial and psychological information, this study provides deeper insights on the interrelatedness of broad aspects of women’s working lives and their career transitions. Second, this study adds a non-Western perspective that reflects Korea’s unique social and cultural characteristics and business environment. Previous research has primarily examined samples in Western countries where the most prestigious research journals are published. Third, this study employs a career history method that portrays the multiple circumstances and root causes of career transitions and does not regard a career transition as a single event. Prior research has rarely explored these cumulative effects of career transitions on an individual’s mobility and coping patterns related to career transitions (Sullivan and Al Ariss, 2019).

## 2 A context-sensitive women’s career: career decision tree model

Women’s career dynamics and underlying complexity cannot be fully explained with an approach that advocates objective rationality that is detached from one’s surroundings. Women’s careers are situated within societally imposed responsibilities (Hjálmsdóttir and Bjarnadóttir, 2021) unlike men’s career decisions that are rather logical and self-centered. Diverse studies on working women have highlighted the impact of different gender role expectations. These studies hve shed light on women’s emotional exhaustion and burnout (Aldossari and Chaudhry, 2021; Gewin, 2021), work-life conflict (Graham et al., 2021; Hjálmsdóttir and Bjarnadóttir, 2021), and lower work performance (Parlak et al., 2021).

Thus, scholars have called for systematic thinking about the dynamics of various influencing factors given the organizational, political, economic, and cultural context (McKie et al., 2013). Bian and Wang (2019) recently proposed a new career decision model to synthesize multi-level influences on women’s career decisions based on an integrative literature review of papers published in the past two decades on women’s career interruptions. CDTM provides a symbolic visual image of a tree consisting of the soil, trunk, branches, twigs, leaves, and fruit to highlight the complexity of the realities and relationships embedded in women’s careers. The metaphoric theme of soil represents the societal culture and norms that affect gender expectations or stereotypes. Soil is critical for the growth of the career decision tree as it provides an environment that affects the other parts of the tree. The trunk symbolizes the national family policies that hold the crown and add strength to the tree. National policies affect a macro labor market that could disadvantage women (Yu, 2006). Each branch of the tree symbolizes a specific industry or occupation within the given context. Twigs are connected to the branches to represent organizations where women actually work in different industries or occupations. The leaves represent individuals’ life events in both personal and professional domains. Last, flowers represent the final career outcomes.

In the same vein, numerous researchers have stressed that such life experiences and career trajectories need to be explored by considering the contexts of different cultures (Kim et al., 2019). For example, Cho et al. (2016, 2019) indicated that women’s career paths in a Confucian culture, particularly in Korea, are heavily influenced by a strongly hierarchical organizational structure, combined with a military culture. In addition, Brinton and Oh’s (2019) study on women in East Asian contexts highlighted the challenges that women face in pursuing full-time employment after childbirth due to strict norms surrounding women’s domestic roles. Given the significance of the socio-cultural influence, this research employs the CDTM as a conceptual framework to identify the hidden clues about the reasons and underlying motives of Korean women’s career transitions. We contend that these reasons and motives are impacted by socially constructed factors within the Korean context.

## 3 Research context

Since the Korean War in the 1950s, along with fast economic development, Korean women’s educational and economic status have dramatically improved. However, the quality of women’s employment in the labor market is still concerning due to the gender wage gap, a high ratio of women engaging in contingent work, and interrupted careers. Typically, women’s economic participation declines during typical childbirth and childrearing years and rises again later in their late 50s (Statistics Korea, 2020), showing a M-shape pattern. The opportunities are also limited for a decent job that accommodates career-interrupted women’s preferences, such as flexible working hours or part-time work, and a work-family balance (Lee and Shin, 2021).

Even in larger organizations, Korean women’s working life is challenging. Korean workers (men and women) spend the longest hours at work (OECD, 2021) while Korean men spend the second shortest amount of time doing house chores (OECD, 2020). This disparity symbolically highlights Korean women workers’ burden inside and outside of work. Korean organizations’ strict management practices are also worth noting. Although the Korean government and organizations have advocated for family-friendly and work-life balance policies, such practices are not yet effective in reality (Kang et al., 2020; Yoo, 2022). Under these circumstances, women need to fully devote themselves to work by leaving their family and personal lives behind or they must quit and leave the workplace. There seems to be no in-between option.

Korea has a unique cultural background that significantly affects women’s lives at home and at work. The cultural characteristics mainly come from Confucius and collectivistic traditions. Hierarchy and strong male dominance in personal and social relationships are still prevalent in Korean society based on the long history of Confucius traditions (Cho et al., 2016; Kim et al., 2018; Brinton and Oh, 2019). Despite some progress, expectations for women’s roles have not significantly changed. For example, they are still the main (and often only) caregivers for children, husbands, parents, and parents-in-law even for working mothers with full-time jobs. Childrearing is still considered the most important job of married women and is a major reason for Korean working mothers’ career interruptions according to the national statistics.

The other main cultural tradition in Korea is collectivism, which impacts many dimensions of Korean life (Hofstede and Minkov, 2010). Individuals who embrace collectivist values have a strong sense of belonging to their reference or identity groups, and they try to be in harmony with others in the group. The sense of belonging has been reinforced by the seniority-based, patriarchal culture in Korea (Han et al., 2018). When making critical decisions, Koreans may not feel comfortable confronting or disagreeing with close others within their reference groups such as parents, husbands, and direct family members (Kim et al., 2019).

Korean research on women’s careers has focused on three key features. One distinctive feature of career transition studies on Korean women focuses on how to enhance women’s employability, particularly for those with interrupted careers. Han and Lee (2019) found that career-interruption and reemployment were the most prevalent topics in career-related research published from 1999 to 2017. Along with the M-shape economic participation rate, research has paid considerable attention to the Korean government’s policy to help support women’s career interruptions. Research has also highlighted the challenges of women’s working lives in Korean organizations (Choi and Park, 2014; Jung and Cho, 2020) which have often led to career transitions including turnover. Scholars have reported that Korean women employees suffer from discrimination in employment practices, wages, and job retention in both public sectors and private companies. Lastly, many research studies have focused on Korean women’s work-life balance issues (Kang et al., 2020; Yoo, 2022). The findings have shown that even among Korean dual-earner couples, women have more difficulty maintaining a work-life balance than men as they take on more responsibilities in the family as the primary caregiver. Specifically, the traditional gender role ideology means that Korean women face more challenges in terms of their quality of life at home and at work (Yoo, 2022). The stereotype of gender roles negatively affects women’s perceptions of their career development outside of the family domain, such as compromising their career success in a company or giving up opportunities for promotions so they can fulfill their family responsibilities (Cho et al., 2015).

Overall, most Korean literature on women’s careers has tended to focus on objective career aspects using relatively large quantitative data. Relevant national and organizational policies have also been explored from a birds-eye view. However, Korean women’s career transitions have seldom been deeply explored with rich descriptions. Even in studies exploring the personal stories of Korean women workers (Cho et al., 2015, 2016, 2017, 2019), the samples have been limited to women with highly successful careers.

## 4 Research methods

This study employed an interpretive paradigm and adopted a basic qualitative research design called a generic or interpretive qualitative research design (Merriam and Tisdell, 2016). This design allowed us to directly interact with participants to comprehend their contexts and lived experiences to show how women manage their career paths (Creswell and Poth, 2018) based on our assumption of socially constructed career behaviors. We also adopted a career history method to collect participants’ career data. Using the career history method, we asked participants to describe their individual career transitions related to the dynamic interplay between their life and the career behaviors embedded in their personal career history. The advantage is that we could gather contextual information on their career transition process and their subjective interpretations of their career experience considering their life situations (Savickas, 2013), which may not be evident from objective career factors such as organizational status, pay, and power (Daniels, 2019; Barnard et al., 2021).

### 4.1 Participants

Participants were recruited through publicly accessible social networks in a large women’s university in Korea. The alumni of the school actively engage in social activities and promote career achievement thanks to the school’s long history and prestigious reputation. This alumni network was suitable to recruit potential participants with rich career histories. After initially contacting the alumni groups and graduate school programs, groups that agreed to help recruit participants posted the recruitment notice on their group websites or social networks. The interview participants could be in any career stage with a work history within the past 10 years even if they experienced career interruptions. We excluded women who had never worked for a living (economic gain), such as older stay-at-home mothers, and those who had not worked for the past 10 years. Although shorter career interruptions are common in Korea, for the purposes of this study, a career break longer than 10 years was considered too long to return to work. We received 35 responses from women who agreed to participate in an in-depth, one-on-one interview about their career transition experiences.

As shown in Table 1, the ages of the participants ranged from 24 to 58, with an average age of 39.4. In terms of education level, 66.7% of the participants had a master’s degree or higher, which means that this was a highly educated group. Their high education level was likely due to the characteristics of the recruitment site (i.e., alumni from a women’s college). Among the participants, three women were in their 20s and two were in their 30s. These participants were relatively young and unmarried. More than two-thirds of the women were married. Considering the average age of marriage (women 31.3 and men 33.7) (Korean Statistical Information Service, 2023), the sample represents the national average. About one-third (28.6%) of the women had no children, with an average of 1.14 children for all participants, which is slightly higher than the national average of 1.1—the lowest in the world (UNFPA, 2021). In terms of current employment status, 34.3% held a regular job while 37.1% held a contingent job. In addition, 17.1% owned their own business, and 11.4% were experiencing a career interruption (i.e., unemployed, housewife, or student) at the time of the interview.

**Table 1:** The characteristics of the participants^a^.

No.	Age	Educational level	Marital status	Number of children	Current position	Work status
1	24	Bachelor’s	Single	0	Nurse	Regular worker
2	25	Master’s	Single	0	Contract researcher	Irregular worker
3	27	Bachelor’s	Single	0	Office worker	Regular worker
4	28	Bachelor’s	Married	0	Business English instructor	Irregular worker
5	29	Bachelor’s	Married	0	Office worker	Regular worker
6	30	Master’s	Married	1	Housewife (previous consultant)	Regular = > Unemployed
7	30	Master’s	Married	1	Educational social worker	Irregular
8	31	Bachelor’s	Single	0	Graduate student (previous part-time worker)	Irregular= > Unemployed
9	33	Master’s	Single	0	Contract researcher	Irregular worker
10	33	Bachelor’s	Married	2	Office worker	Regular= > Irregular
11	33	Bachelor’s	Married	1	Office worker	Regular= > Irregular
12	34	Master’s	Married	1	Office worker	Regular worker
13	35	Master’s	Married	2	Housewife (previous contract office worker)	Irregular= > Unemployed
14	35	Bachelor’s	Married	1	Housewife (previous designer)	Regular = > Irregular= > Unemployed
15	36	Master’s	Married	1	Office worker	Irregular= > Regular
16	36	Master’s	Married	1	Contract researcher	Irregular = > Regular = > Irregular
17	36	Master’s	Married	1	Laboratory administrative assistant	Regular = > Irregular
18	39	Bachelor’s	Married	1	Office worker	Regular
19	40	Master’s	Single	0	Assistant administrator	Irregular
20	40	Bachelor’s	Married	3+	Teacher	Regular
21	41	Master’s	Single	0	Business owner	Irregular= > Regular= > Self-ownership
22	42	Master’s	Married	2	Contract teacher	Regular = > Irregular
23	46	Master’s	Married	2	Extra-curriculum program tutor	Irregular= > Regular= > Irregular
24	48	Bachelor’s	Married	2	Office worker	Regular
25	51	Doctoral	Married	1	College instructor	Regular = > Self-ownership= > Irregular
26	52	Master’s	Married	2	Business owner	Regular = > Self-ownership
27	52	Doctoral	Single	1	Business owner	Regular = > Irregular= > self-ownership
28	52	Master’s	Single	1	Business owner	Irregular = > Self-ownership
29	56	Master’s	Married	2	Business owner	Regular = > Irregular= > Self-ownership
30	48	Master’s	Married	2	Office worker	Regular worker
31	46	Bachelor’s	Married	2	Extra-curriculum program tutor	Irregular worker
32	50	Bachelor’s	Married	3+	Teacher	Regular worker
33	42	Bachelor’s	Married	2	Business owner	Regular = > Self-ownership
34	58	Master’s	Married	2	Teacher	Regular = > Irregular= > Regular
35	41	Bachelor’s	Single	0	Office worker	Regular

a The list of participants is sorted by interview order.

### 4.2 Data collection and analysis procedures

We conducted one-on-one interviews via phone or Zoom (audio function) due to Covid-19 restrictions during the data collection period from July to October 2020. We adopted a purposive sampling method to recruit participants. After potential participants expressed interest in the study, they were screened for inclusion. We also informed them of their rights and ensured confidentiality. Prior to the interview session for each of the 35 participants, we collected information via email or SNS on their work and employment history including the starting and ending date of each job that they had since college graduation, the organization/company names and locations, job titles, and any additional details provided. We reviewed their work history before each interview and then conducted an in-depth, semi-structured interview by phone or Zoom using only voice mode for both phone and Zoom. Interviews lasted between 1 and 2 h in Korean and were recorded with the participants’ permission and later transcribed verbatim.

The one-on-one interviews allowed researchers to hear each woman’s individual perspective on their thoughts and behaviors related to their individual career paths. The individual interviews also enabled the researchers to ask follow-up questions to explore new issues related to contextual information (Boyce and Neale, 2006). The basic format of the interviews included nine questions about their school-to-work process, such as their first job experience, subsequent career transitions, turnovers and layoffs, career transition-related events or episodes, personal feelings associated with their decisions, and influencing factors that affected their career transitions. The information on their work history was also utilized as the basis for asking follow-up questions during the interviews. For example, if an interviewee had a short-term work experience, we asked whether the position was a contract-based or full-time regular position. When an interviewee included information about a turnover from a promising position, we asked about her life circumstances that might have affected her decision. For example, we sometimes asked whether the interviewee started her own family when she had a career interruption.

A thematic analysis following Braun and Clarke (2013) was used to identify common themes in the dataset. First, the first two authors read and reread all the transcripts to become familiar with the stories. In addition, the first author added many notes to the transcripts to capture early ideas. Then initial codes for interesting features across the entire dataset were created including the following: unprepared for the first job search, parents’ extended interference, physical health issues, emotional stress, voluntary turnover, changes in career orientation, positive or negative family influence, initial focus on external/objective aspects of the career, longing for work-life balance, child rearing, and unexpected life events. The authors then categorized the initial codes into possible themes along with quotes and alignment between the themes, and the coded quotes were checked. In this process, the first two authors shared the codes and exchanged opinions and readjusted the codes and themes in a collaborative way. Definitions and headings for each theme were refined by the first author and finally representative quotes for each theme were gathered by the second author. To enhance the trustworthiness of the data, we conducted a member check to prevent distortion when interpreting the responses (Creswell and Poth, 2018). After completing the initial data analysis, three participants who initially (during the interviews) showed interest in member checking were invited to review the summary of our findings. One of the comments was a clarification about how internal and external factors that affected their career transitions were different. Other comments were mostly clarification questions about the description of the summary. For instance, one participant asked about “changes in priority” in the initial analysis. Thus, we explained what we meant by priority in the transcript and the changes in priority that we observed in the interview transcript.

## 5 Findings

Overall, participants’ careers were interrupted with many short breaks, but some also experienced long breaks. Among the hundreds of career moves participants reported, almost all participants said their career moves were based on a voluntary decision except for two who experienced forced layoffs. Five separate themes emerged from the interview data analysis. However, we acknowledge that the complete stories of the participants’ experiences and relevant themes are better understood in a holistic way.

### 5.1 Unresolved early career interests and expectations

The participants’ career transitions were based on unresolved career interests and expectations. Sixteen participants were not happy with their work and wanted to pursue a better fitting job, particularly in their early career selection process. When asked about their motivation in selecting their early career, they gave three reasons. The first reason was that it was a typical job that they could easily find in their field based on their college major. For example, a college instructor described the easy track: *“I have only been doing art since I was a kid and it became my major in college …And I started individual artwork after graduating. I did not think about anything else.”* The second reason was that they selected the job/workplace because it was generally considered desirable, ideal, or recommended by someone close to them. One participant remarked: *“I did not seriously consider whether it fits who I am. My first job looked good to others. Bank employees were well perceived, in general, because you can go home on time [not like other companies]. But, to me, it was hell.”* The third reason was that they randomly found a job opportunity and were lucky to be hired. For example, an office worker said: *“After I complained about my job while we were eating, my friend said she would refer me to her acquaintance who was in a management position in the broadcasting industry.”*

A potential underlying issue related to these unresolved early career interests and expectations was a lack of effective career exploration when they were growing up or in college. One participant said: *“I started to think about what I like and who I am actually after I got married. Before that, I did not think about it much…I just tried different things just to find my path.”* Six participants did not initially select a college major considering who they were or their unique interests. Particularly older participants’ ideas about their careers were somewhat vague when they were young; thus, their preparation for their future careers was not strategic. Consequently, their unresolved early career interests seemed to result in unenjoyable early jobs, which led to a career transition. Their career paths appeared to be a process of searching for their true career interests and adjusting to their life’s circumstances at the same time.

*From the beginning, I did not have any interest in education. I had wanted to study music since I was little. But my parents said no. My mother said, “Now, study preschool education and get a teacher’s license first. After that, you can do whatever you want.” Well, the conversation was flowing like this. I had no choice but to study preschool education, become a teacher, and I had no choice but to maintain that job until I had my second child. Then, after a break, I made a transition to music.*

Participants with unresolved career interests and expectations faced other personal issues such as health problems and family issues that tended to more easily dismantle their careers. In other words, they already did not like their job so when another issue arose, it decisively prompted them to make a serious career decision. This trend was also observed in other themes.

### 5.2 Personal health problems

One of the most troubling reasons for our participants’ career transitions was personal health problems. Short career breaks were especially prompted by personal health problems that were primarily caused by harsh working conditions. Participants made numerous statements about their physical health (e.g., fatigue, stomach troubles) and mental health issues (e.g., burnout, depression). They used negative words such as stress (29 times), depression (12 times), and bad health condition (8 times). Two women even mentioned panic attacks due to work-related stress. Sixteen participants made similar statements referring to personal health problems such as *“The work was so hard. I went to work very early. Lots of meetings, Then, I got sick. Very sick… Even if I went to see a doctor for more than a month, my body condition did not get any better.”* They mostly mentioned mental stress or burnout but also physical illness as reported in the next excerpt: *“I became so tired mentally and physically… too much stress. And, it made me literally sick.”*

This trend was particularly prevalent among participants who were younger than 40. However, it did not seem to be associated with marital status even though the general perception in Korea is that married women have a heavier dual burden.

*In the early days of working at the hospital, I [nurse] often could not eat or go to the bathroom. It was the culture for the medical staff there. Of course, nobody said I could not go to the bathroom, but when you are busy, you forget. You can learn in biology class that when someone is in an emergency, the bladder relaxes, and you just forget to go to the bathroom. I could not go to the bathroom even though I had my period… I felt my health deteriorated.*

### 5.3 Family issues

Thirteen participants with children reported that family issues, particularly childrearing, was the reason for their career transition. However, it should be noted that this reason was mentioned less frequently than personal health problems. Furthermore, as a single factor, it rarely resulted in a career transition but was particularly influential when combined with other reasons such as feeling lost in their career, or a bad business or market situation in addition to personal health issues. Childrearing seemed to be a *de facto* trigger to initiate or reinforce their career change decision. Although fifteen married participants in their mid- or late-career stage brought up getting married, family relocation, or their husbands’ influence as a reason for a career transition, half of them cited it in combination with other complicated issues at work.

*When I heard about my child’s anxiety from the psychiatrist… I realized there is no reason to keep my career… Frankly speaking, I felt relieved rather than being disappointed. I thought “OK. This is a valid reason to quit my job”… My business was also running down. I was wondering about how to deal with the situation anyway. Then, with an excuse of my child’s issue, I came to think “Let us close it down.”*

*There were already many serious problems inside and outside. The work was overwhelming, so I could not enjoy it anymore. Around that time, my mother-in-law lost 7 kilograms because she commuted for a long distance between Seoul (my house) and Ulsan (her house) to take care of my child. When her health problems occurred, I felt that I could not keep my job.*

### 5.4 Changes in values and priorities

Thirty-two participants discussed the changes they experienced in their values and priorities along with two obvious trends. Their orientation shifted from being external value-focused in their early career choice to being internal value-focused. In response to a question about their initial career choice, they confessed that they chose their first job because it looked socially good or preferred by others without a deep self-examination of their own career goals and plans, as discussed in the first theme. The “others” included significant others (usually parents) as follows: *“My parents, especially my mother, really liked me to have a stable job in a big company. That is why I ended up in my first workplace. But I realized that I did not enjoy working there at all.”*

Along their career paths, many participants finally learned about themselves and started to be involved in work that corresponded to their desires. Almost half of the participants came to realize the importance of being more honest about who they were. In addition to searching for authenticity, many participants emphasized maintaining a balance between work and life. Younger women were more likely to pursue a work-life balance, and the areas of life included religion, personal hobbies, or dedication to a cause besides their family. Most married participants had a more traditional view of the balance between work and family.

*After college, the important thing was how famous the company was… I cared about only external things like how much I can make, how good the company building is. But, as I worked, I learned that such external things were not important. I needed to find my interests.*

*In the past, I just wanted to build a good career in the hospital to make more money. But now, I realized that my health is also important… I only walked forward until now, but I want to focus more on myself at present. I do not want to pursue money anymore. Now, I think money is not everything.*

### 5.5 Unfriendly labor market and demanding workplace

Indirectly, the participants’ career transitions were highly affected by their working environment. In particular, older women with interrupted careers felt that they were not welcomed nor competent in the Korean labor market while younger women’s career transitions were strongly affected by a demanding work life, which was one of the most obvious trends that stood out in our study. For the former, the challenges included limited decent job opportunities for career interrupted women and unfair treatment such as gender and/or age discrimination in the job market. Participants strongly complained about these factors whenever they attempted to rejoin the workforce after a career break, and their stress impacted their well-being.

*After my second child grew up, I tried to get a job in earnest… Last year, I worked really hard to find a [regular] job. I wanted to return to the broadcasting industry rather than being a freelancer. There are not many positions for middle-aged women whose careers were broken. I just got one job interview after my active search for more than 6 months. I could not even get the job. I felt I had to give up working again.*

*In my 20s, I felt that I was as competent as men when I graduated from college. But, after I gave birth, I became handicapped in the labor market. The only difference is that I became a mom.*

Women who had a job also suffered under the pressure at work. The women frequently mentioned the overwhelming workload as shown in the following excerpt: *“Even if I liked the job, I knew I could not stay long there…I felt I was getting totally exhausted because of too much work. I had no personal life except for work.”* They reported that it was clear that there was no way to meet the expectations of their roles and responsibilities in the workplace and this workload level caused serious damage to their health, which eventually led them to escape from the harsh situation. Additionally, an unfriendly organizational culture toward women (especially mothers) was noted. Frequently, they had no luxury to enjoy a women-friendly organizational policy or assistance programs due to the heavy workload and unfriendly organizational atmosphere. Additional concerns included one-sided communication issues, authoritarian leadership, and conflicts in interpersonal relationships.

*When I went home from work, I usually felt too tired. So, I went to sleep early, and then woke up again in the middle of the night, like 2 or 3 am [to finish up my work] and went to work in the morning. I was overworked without weekends… I felt my life was going to shatter even though I liked the job.*

*Speed, speed, speed. It did not make sense. For example, I was asked to complete a task in two months that normally took four months. I felt overwhelmed… I had to manage not just one task, but 4–5 projects at the same time, which choked up workers… They knew it was impossible but kept pressuring me. It seemed like “show me how competent you are.” I hated this culture.*

*There is a psychological counseling center in the company. I needed to make an appointment to receive the service. But I could not do it within the working hours. I could not even make a phone call because I was too busy to call there.*

*Officially, I had an option of maternity leave, but I could not use it in reality. The atmosphere of my company does not allow it… It is related to my boss’ personal character. I knew she did not like it… I did not even use paid time-off except for three days at maximum even though I really needed it when my daughter was hospitalized. I went to work during the daytime and slept in the hospital [where my child was] at night.*

## 6 Discussion

**Figure 1:**
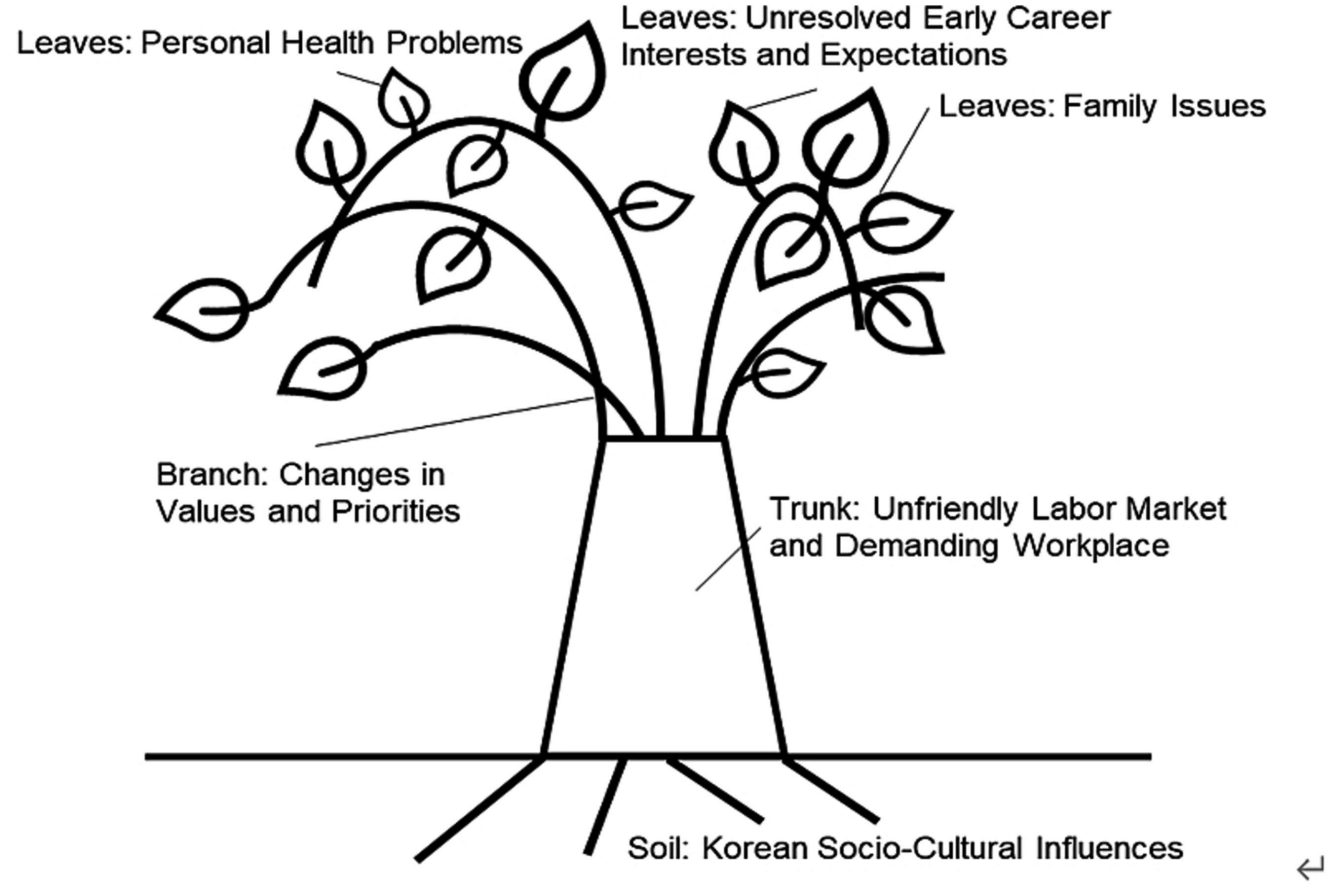
Causes and influencing factor of career transition in Korean women. Adapted from the Career Decision Tree Model [adapted with permission from Bian and Wang (2019, p. 810)].

Korean women workers’ experiences of career transitions are inextricably entwined with the business practices where they work and with their relationships with others as well as with society. Five causes and influencing factors of women’s career transitions can be symbolically presented, as shown in Figure 1. The model was adapted from the Bian and Wang’s (2019, p. 810) original CDTM. Specifically, we built this model based on the Soil, Trunk, Branch (combined with Twigs), and Leaves visual. Since our focus was a career transition as a final product, we omitted Fruit in the original model.

### 6.1 Soil

Soil in CDTM refers to societal culture and norms. In our model, Korea’s Confucius and collectivistic traditions (Cho et al., 2016; Brinton and Oh, 2019) pass from the Soil to the Trunk, Branches, and Leaves. The participants had to handle social expectations as the primary family caretaker given the traditional Korean Confucius culture at home. Their role as caretaker often became a justifiable reason for their career interruption. They seemed to persuade themselves that fulfilling their family’s expectations and needs was a valid excuse for a career transition, but the expectation of women’s roles was not the sole trigger of their career interruption. The findings from the mainstream literature contrast with this finding as fulfilling women’s caretaker roles is a well-known factor of women’s career interruptions (Bian and Wang, 2019). On the surface, Korean women’s reasons seem to be similar, but the drivers under the surface were different and were influenced by the larger social culture.

The women also felt a burden to be a harmonious organizational member even in a harsh workplace. They passively dealt with workplace issues by voluntarily leaving the workplace. Among the hundreds of career transitions reported in our sample, only two were forced turnovers. Under the collective tradition, workers may cautiously avoid noisy conflict in the workplace. Additionally, standing on the Soil, Korean women were not well prepared or strategic about their career choices. Many women turned to what others said or how others viewed them when they were young. After they entered the labor market, they quietly suffocated under the business practices and organizational culture.

### 6.2 Trunk

The Trunk in CDTM represents the country’s family policies. However, Korea’s family policies were not discussed in our study. Family policies exist, but the practical impact was not discussed in the interviews. It may be that Korea’s national policies did not seem to help the women resolve their career issues. Thus, a noticeable discrepancy was found between official regulations and polices and the actual effectiveness of the policies.

The participants mostly attributed their career interruptions to challenges in the macro labor market, and more specifically, to the tight labor market and unfriendly business practices for women. In previous Korean studies, organizational-level meso factors including limited advancement opportunities, discrimination (Powell, 2018; Gauci et al., 2022), and harassment (Fielden and Hunt, 2014; Cortina and Areguin, 2021) have also been reported (Hong and Gye, 2016; Quan and Shim, 2022). Even at the global level, numerous studies have documented that especially working mothers struggle under a gendered organizational atmosphere (Cahusac and Kanji, 2014; Stone and Lovejoy, 2019) where traditionally masculine modes of working are the norm (Noh et al., 2012; Cook et al., 2022).

A unique finding in our study was that Korean organizations’ demanding workload with long working hours made it difficult for women to survive in organizations. Previous studies have also reported time commitment required in Korean companies under the collectivistic culture (Kim and McLean, 2014; Kim et al., 2018; Min and Lee, 2021). It is not unusual for grandparents to raise their grandchildren in their home, and the parents only see the children on weekends because of their busy working hours during the work week (Kim and Chung, 2011). The hierarchical organizational structure, top-down communication, and emphasis on collectivistic organizational behaviors have been cited in the literature (So, 2012; Kim et al., 2021). As a result of these challenges, many of the Korean women in the current study became unhealthy both physically and mentally. These challenging working conditions also function as strong push-out factors that disadvantaged and interrupted women’s careers (Kossek et al., 2017).

### 6.3 Branch

In the original CDTM, a Branch represents a specific industry or occupation (Bian and Wang, 2019). In this study, Korean women’s career transitions spanned across industries and occupations, so the same meaning of Branch is not applied. Instead, we found that common changes in values and priorities represented the Branch that links the Trunk and Leaves, which led to the women’s eventual career transitions. Almost all of the participants (32 participants) mentioned a change in their values and priorities along their career path regardless of the career issues they faced. This Branch bridges the labor market (macro level), organizational culture (meso level), and individual causes (micro level) of career transitions. The participants recalled that what they valued in their early career stages had changed based on their experience in the workplace and changes in their personal lives. These participants initially believed that the meaning of a career was to fulfill their basic needs or gain recognition and rewards. Kim’s (2021) study also found that Korean women’s early failure to listen to their own inner voices instead of the surrounding socio-cultural influences seemed to later intensify their longing to meet their own needs and desires.

### 6.4 Leaves

The Leaves symbolize the direct causes of career transitions. Symbolically, when a participant experienced a career transition, one or more Leaves that were connected to the Branch, Trunk, and Soil fell to the ground. For example, sixteen participants discussed their unresolved career interests and expectations linked to the lack of self-understanding and career preparation, particularly when they transitioned from school to work. This finding is also supported by previous Korean research with a similar population (Kim et al., 2019) of women college students who were less strategic in their early career pursuit compared to their male counterparts (Kim and Lee, 2017). Unlike this finding, most mainstream literature has viewed career transitions as the result of a well-developed understanding of oneself and strong motivation (Sorrentino, 2006; Lent and Brown, 2019; Nakitende, 2019; De Vos et al., 2021).

Personal health problems were also found to be the direct cause of many of the participants’ career transitions. To the best of our knowledge, health problems have not been reported in the research with a sample of general women workers. Given the demanding organizational environment in Korea, many of our participants experienced mental stress and burnout as well as physical illness that required professional medical care. Previous research on psychological difficulties of working mothers has mostly focused on mental health including stress, burnout, anxiety, and depression stemming from their dual roles (Lee et al., 2017; Thapa, 2019). However, the current study goes beyond mental health and revealed both psychological and physical health issues as direct strong influencing factors that led to a career transition.

Family-related reasons mostly included childbirth and childrearing, which confirms prior research on women’s career interruptions (Cabrera, 2007; Bian and Wang, 2019). However, the participants’ stories uncovered the underlying causes of the family-related reasons that led to the women’s career transitions. Because they were already under stress at work or struggling with other issues, these underlying unresolved issues seemed to be combined with the surface family-related issues. A family-related issue was frequently a legitimate excuse to justify their career decision when they were unsatisfied with their current jobs. Even in a Western context, Summers et al. (2014) and Boushey (2008) cautioned that explaining the need for childcare as a sole cause of women’s opt-out decision reinforces stereotypes about women’s roles. Thus, it may be too quick or imprudent to claim that women around the world leave a job solely due to work–family conflict. This argument could be strengthened by viewing job transitions as a complex decision-making process that includes resolving work–family conflicts as well as other factors (Radcliffe and Cassell, 2014; Thomason, 2021).

## 7 Implications for research and practice and limitations

The findings of this study provide insights for future research on women workers in organizations. First, this study highlights the importance of considering culture when examining women’s careers and career transitions (Adya, 2008; Mate et al., 2019). In particular, this study identified subtle differences in the causes of career moves within the social-cultural context of Korean women’s career histories. Future culture-specific research on women’s career development is encouraged using a qualitative approach that deeply explores the underlying career motives, interests, and values of women by understanding the immediate and long-term career transition process and associated factors. For example, a narrative research approach to explore women’s career development is useful because it allows participants to share their lived experiences through rich discourse, as shown in Bowles et al. (2019), Chinyamurindi (2016) and Cho et al. (2016).

The findings also help us understand the deeper reasons and influencing factors of women’s career transitions. It would be interesting to examine how these factors are related in women’s career journeys. For example, how do family-related issues positively contribute to women’s career development when organizational issues act as push-out forces that often lead to women’s career interruptions? What family dynamics including influence from family members result in women workers’ career transition decisions? Are women’s reactions to family issues different depending on their socio-cultural backgrounds? These potential research questions should be explored further. The answers can contribute to theory-building for a deeper understanding of women’s career transitions in organizations and the cultural implications.

Another implication is the need to investigate the changing patterns in career values and priorities that were identified in this study. Since this trend has also been reported in previous Korean studies (Kim and McLean, 2008; Kim et al., 2019; Kim, 2021), it deserves further examination. After controlling for potential intervening variables such as age, a quantitative study may provide empirical evidence to validate whether the career patterns of being influenced by external factors to focusing on internal factors is solely due to gaining career experience. Changing patterns of women’s subjective career orientation could be examined using grounded theory to help develop a new theory. Researchers could also explore whether this trend is universal across the globe.

In terms of practice, considering that older generations recounted their poorly developed career concepts and rather disorganized career preparation prior to their school-to-work transition, organizations can strengthen career development programs so young women employees solve potential career issues in the workplace. It is also necessary to maintain training programs to help them prepare for future career transitions. Managers and human resources can also play an important role to help women workers effectively deal with future developmental issues.

A demanding workload and unfriendly business practices for women in Korean organizations have been reported in many previous studies (Noh et al., 2012; Cho et al., 2016; Kim et al., 2018; Lee and Shin, 2021). However, few previous studies have focused on how both mental health and physical health impact career decisions. We paid special attention to our participants’ physical and mental health issues because they can cause a serious burden for the family and society beyond the individual. Strong interventions are needed to help women workers cope with stress in the workplace. Many participants were unable to use the available counseling and policies in the organization because of their busy schedules, and others suffered physical illnesses due to work pressure. These experiences highlight a critical need for organizations to monitor how their policies are implemented. Policy makers must also promote changes in women-friendly policies, assistance programs, and benefits at the national level.

Previous studies have highlighted the discrepancy in Korean women’s lives between the public sector and private sector (Cho et al., 2016). Changes in regulations, policies, programs, and accommodations in the public sector have not necessarily impacted women’s private lives. By acknowledging social expectations on women in Korea, researchers must delve deeper into individual women’s working lives to help change the cultural norms in the workplace that affect women’s private lives. Education is also needed for corporate leaders. Changing how leadership views and treats women in the workplace is critical because management’s vision has a significant impact on organizations (Ford and Ford, 2012; Elliott, 2022). At the societal level, the significance of women workers’ career issues needs to be discussed publicly and various solutions need to be explored to address discrimination against women (Glass and Cook, 2016).

Despite the theoretical and practical implications, this study has limitations that should be considered for future research. First, the participants in this study were highly educated due to the recruitment site. Because education level is a meaningful variable when researching career phenomena in Korea (Chang et al., 2010; Na and Chang, 2019), the findings of this study could have been attributed to these participants’ demographics. Future studies need to examine if women with less education have similar experiences. It is likely that they face even harsher challenges. Second, it was impossible to capture all the career transitions in the participants’ career paths because their careers were like a complicated “zigzag” pattern (Gersick and Kram, 2002, p. 31). Thus, their part-time and short-term work experiences may not have been fully considered in the data collection and analysis. The participants may have also been unable or unwilling to recall details of all their transitions. Future research that fully captures even minor career moves and overcomes the inevitable omission of data would provide a more comprehensive understanding of women’s career transitions.

## 8 Conclusion

Careers are increasingly chaotic, and women’s careers are particularly susceptible to social factors and life events (Elley-Brown et al., 2018; Jogulu and Franken, 2022). Given that women workers’ career experiences have not been explored extensively through a cultural lens in the mainstream literature (Kossek et al., 2017), the purpose of this study was to understand Korean women workers’ career transitions using a qualitative approach. On the surface, Korean women workers’ career transitions are similar to those of other women in global research showing multi-level issues and factors. However, instead of acting as self-agents with a strong career vision and deliberate planning and preparation (Shapiro et al., 2008) as discussed in most Western literature, Korean women workers often appeared to be pushed out of the workplace with no other option because they were too physically sick and mentally stressed to work. Unique characteristics of Korea’s socio-cultural traditions and organizational business practices may explain these differences. As such, to better understand women’s career behaviors, it would be beneficial to conduct more culture-specific research on women’s careers by highlighting the underlying psychological and contextual challenges associated with women’s careers.

## Data availability statement

The raw data supporting the conclusions of this article will be made available by the authors, without undue reservation.

## Ethics statement

The studies involving humans were approved by Ewha Womans University Institutional Review Board. The studies were conducted in accordance with the local legislation and institutional requirements. Written informed consent for participation was not required for this study in accordance with the national legislation and the institutional requirements.

## Author contributions

NK: Writing – original draft. KK: Writing – original draft. PB: Writing – review & editing. All authors made a substantial, direct and intellectual contribution to the work, and approved it for publication.
